# Experience with tafamidis in peritoneal dialysis for a patient diagnosed with transthyretin cardiac amyloidosis

**DOI:** 10.1093/ckj/sfae233

**Published:** 2024-07-29

**Authors:** Diego López Fazlic, Samuel Abrante García, Micaela Gerard, Edduin Martín Izquierdo, Alejandro Alonso Bethencourt, Luca Vannini, Celestino Hernández García, Manuel Macía Heras

**Affiliations:** Cardiology Department, Nuestra Señora de la Candelaria University Hospital, Santa Cruz de Tenerife, Tenerife, Spain; Nephrology Department, Nuestra Señora de la Candelaria University Hospital, Santa Cruz de Tenerife, Tenerife, Spain; Nephrology Department, Nuestra Señora de la Candelaria University Hospital, Santa Cruz de Tenerife, Tenerife, Spain; Nephrology Department, Nuestra Señora de la Candelaria University Hospital, Santa Cruz de Tenerife, Tenerife, Spain; Nephrology Department, Nuestra Señora de la Candelaria University Hospital, Santa Cruz de Tenerife, Tenerife, Spain; Cardiology Department, Nuestra Señora de la Candelaria University Hospital, Santa Cruz de Tenerife, Tenerife, Spain; Cardiology Department, Nuestra Señora de la Candelaria University Hospital, Santa Cruz de Tenerife, Tenerife, Spain; Nephrology Department, Nuestra Señora de la Candelaria University Hospital, Santa Cruz de Tenerife, Tenerife, Spain

**Keywords:** peritoneal dialysis, tafamidis, transthyretin amyloidosis

## Abstract

Cardiac amyloidosis is a cardiomyopathy resulting from the extracellular deposition of proteins such as transthyretin (TTR). We present the case of a 72-year-old male with hereditary cardiac amyloidosis. After confirming the diagnosis, tafamidis, a TTR stabilizer, was administered. Remarkably, tafamidis, when coupled with peritoneal dialysis for chronic kidney disease, maintained stability in both cardiac and renal functions. Previous studies have demonstrated the efficacy of tafamidis in reducing all-cause mortality and cardiovascular hospitalizations, although its use in severe renal failure lacks specific evaluation. This case suggests a potential application of tafamidis in moderate–severe kidney disease, emphasizing the need for further research in this population.

## INTRODUCTION

Cardiac amyloidosis is an infiltrative disease caused by the extracellular deposition of proteins. Transthyretin (TTR) produces one of the most common forms of cardiac amyloidosis [1, 2]. The incidence of cardiac amyloidosis has increased in the last decade due to improved diagnostic techniques, accompanied by new therapeutic strategies that have been shown to improve the survival and quality of life of these patients. However, a percentage of individuals affected by this disease still cannot fully benefit from these therapies due to the lack of evidence in patients with severe kidney disease.

## CASE REPORT

The patient is a 72-year-old male with a history of hypertension, type 2 diabetes mellitus with microvascular involvement, chronic kidney disease (CKD), dyslipidaemia, polymyalgia rheumatica and intervention for bilateral carpal tunnel syndrome.

**Figure 1:**
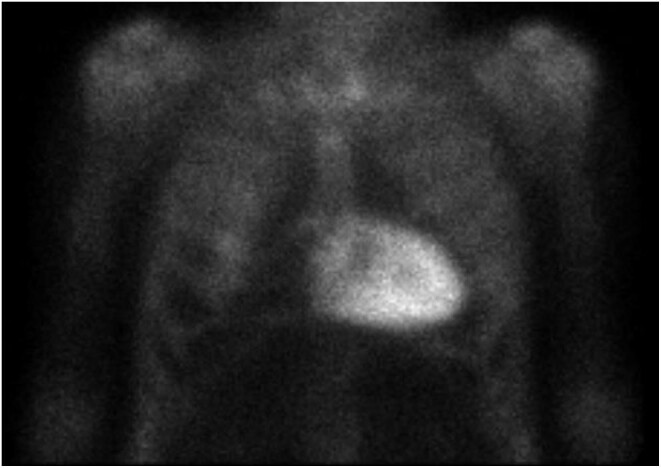
99mTc-DPD scintigraphy showing high- grade cardiac uptake.

He arrived at the emergency department in January 2021 with central chest pain associated with vegetative symptoms. A transthoracic echocardiogram revealed systolic function at the lower end of the normal range, without segmental disorders of contractility, and mild biauricular dilation. Giving the suspicion of infiltrative cardiomyopathy. In May 2021, a 99mTc-DPD scintigraphy (Fig. 1) was performed, showing ‘increased myocardial uptake’ of high intensity (Perugini grade 3/3), demonstrating a scintigraphic pattern compatible with cardiac amyloidosis due to TTR (ATTR).

A genetic study was requested to distinguish between hereditary ATTR (ATTRh) and wild-type ATTR (ATTRwt), revealing a probable pathogenic mutation c.424G>A (p.Val142Ile) in heterozygosity in the TTR gene, thus confirming the diagnosis of ATTRh.

Treatment options targeting the disease were proposed, such as tafamidis and patisiran. However, the latter was initially ruled out due to deterioration in kidney function. In May 2022, the decision was made to initiate treatment with tafamidis 20 mg, a dose lower than recommended. Until then, the patient had presented with up to four more episodes of hypervolaemic decompensation of heart failure, with N-terminal pro-B-type natriuretic peptide (NT-proBNP) levels of 17 000 pg/ml, which led to his hospital admission and subsequent outpatient diuretic treatment.

From a renal perspective, the patient presents persistent alteration of renal function with baseline creatinine ≈1.7–1.9 mg/dl, proteinuria 1.7 g/24 h and glomerular filtration rate ≈28 ml/min/1.73 m^2^.

After the introduction of tafamidis, the patient remained stable for up to a year without experiencing new episodes of heart failure decompensation. Baseline NT-proBNP levels stayed consistent at ≈10 000 pg/ml; however, he presented symptoms of overload such as dyspnoea and oedema that were partially controlled at home.

In May 2023 he again presented with congestion and dyspnoea refractory to diuretics, with worsening of his kidney disease. Therefore it was decided to start the patient on peritoneal dialysis.

Currently he is being treated with tafamidis 20 mg and continuous ambulatory peritoneal dialysis (CAPD). The patient presents good evolution and stability from a nephrologic point of view, with effective peritoneal dialysis, preserved residual renal function and urinary volumes in 24 h of ≈900–1200 ml. There have been no new admissions due to decompensated heart failure after starting CAPD.

## DISCUSSION

Tafamidis has not been specifically evaluated in a population with impaired renal function or in renal replacement therapy, therefore its use is not recommended in these patients. However, pharmacokinetic estimates did not indicate significant differences between different renal stages. Among the adverse effects described from a renal perspective in the study mentioned above, a high percentage of acute kidney failure was demonstrated, which paradoxically was observed more frequently with the tafamidis 20 mg formulation (10.2% of cases) versus tafamidis 80 mg (7.4% of cases) [1, 2]. In the ATTR-ACT study (NCT01994889) there was a significant reduction in the increase in NT-proBNP over time with tafamidis 80 mg compared with placebo. Additionally, the reduction in the increase in NT-proBNP was significantly greater with tafamidis 80 mg than with tafamidis 20 mg. This may suggest that patients treated with doses of 20 mg had worse control of heart failure with consequent deterioration of renal function [2].

In the Long-Term Safety of Tafamidis in Subjects With Transthyretin Cardiomyopathy study (NCT02791230), patients who completed the ATTR-ACT were eligible for enrolment in a long-term extension (LTE) study. In this study, the incidence of adverse events was similar or lower than that with pooled tafamidis (80 and 20 mg) or placebo in the ATTR-ACT. In the LTE study, 18.4% of randomized patients presented an acute kidney injury as an adverse event [3].

The treatment of amyloidosis by TTR aims to support complications, manage heart failure and prevent disease progression [1, 3]. The most used drug is patisiran, demonstrating up to an 80% reduction in TTR production [4]. However, its use is not recommended in patients with severe renal failure or end-stage renal disease, due to insufficient research in this subpopulation of those affected by ATTRh. Therefore, tafamidis is presented as a treatment option in the above case, as there is no clear recommendation and/or contraindication regarding its use in advanced-stage kidney patients.

In this case, the initiation of CAPD has been the main factor to avoid new decompensations, supported by a progression stabilizer such as treatment with tafamidis.

Although there are no safety studies in a renal cohort that can support the use of tafamidis in patients with severe renal failure, the description of this case could serve as a basis, at least in our centre, for the use of this drug in patients with moderate–severe kidney disease.

## CONCLUSION

In our patient, the use of tafamidis under a PD regimen has not led to a worsening of residual renal function. Furthermore, since their joint application, there have been no new episodes of heart failure decompensation.
